# The complete chloroplast genome of *Euscaphis japonica* (Thunb.) Kanitz (Staphyleaceae) isolated in Korea

**DOI:** 10.1080/23802359.2020.1835571

**Published:** 2020-11-11

**Authors:** Sang-Hun Oh, Jongsun Park

**Affiliations:** a Department of Biology, Daejeon University, Daejeon, Republic of Korea; b InfoBoss Inc., Seoul, Republic of Korea; c InfoBoss Research Center, Seoul, Republic of Korea

**Keywords:** Crossosomatales, *Euscaphis japonica*, intraspecific variations, Jeju Island, Korea

## Abstract

The complete chloroplast genome of *Euscaphis japonica* (Thunb.) Kanitz isolated in Korea is 160,606 bp long and has four subregions: 89,232 bp of large single-copy and 18,734 bp of small single-copy regions are separated by 26,320 bp of inverted repeat regions including 129 genes (84 CDS, 8 rRNAs, and 37 tRNAs) and three pseudogenes. There were 424 SNPs and 809 INDELs compared with the Chinese *E*. *japonica*, useful to develop markers for phylogeographic study of the species. Phylogenetic trees show that *E*. *japonica*, representing Crossosomatales, is nested within the Malvids clade, confirming previous studies.

*Euscaphis japonica* (Thunb.) Kanitz, commonly called the Korean sweetheart tree, is a deciduous tree distributed in warm temperate regions of Korea, China, Japan, and Taiwan, and widely cultivated in garden for its showy fruits. It is characterized by having follicular fruits and arillate seeds in Staphyleaceae (Li et al. 2008), a member of the recently re-circumscribed angiosperm order, Crossosomatales (Oh and Potter 2006). A recent phylogenetic analysis of angiosperms placed Crossosomatales as sister to a large clade of Picramniales, Malvales, Brassicales, Huerteales, and Sapindales (Soltis et al. 2011). We determined complete chloroplast genome of *E. japonica* to confirm the phylogenetic position of Crossosomatales and to investigate intraspecific variations (Xiang et al. 2019).

Total DNA of *E. japonica* collected on Jeju Island in Korea was extracted from fresh leaves by using a DNeasy Plant Mini Kit (QIAGEN, Hilden, Germany). The voucher specimen was deposited in the herbarium of Daejeon University (TUT) (*Oh 7720*; 33°17′24.35″N, 126°26′34.41″E). Genome sequencing and *de novo* assembly were done by the method described in Park et al. (2019b). Genome annotation was based on *E. japonica* chloroplast genome (MN159078) using Geneious R11 v11.0.5 (Biomatters Ltd, Auckland, New Zealand).

Chloroplast genome of *E. japonica* (GenBank accession is MT702885) is 160,606 bp (GC ratio is 37.3%) and has four subregions: 89,232 bp of large single-copy (LSC; 35.4%) and 18,734 bp of small single-copy (SSC; 31.6%) regions are separated by 26,320 bp of inverted repeat (IR; 42.7%). It contains 129 genes (84 protein-coding genes, 8 rRNAs, and 36 tRNAs) and three pseudogenes; 17 genes (six protein-coding genes, four rRNAs, and seven tRNAs) and one pseudogene are duplicated in IR regions.

Based on pairwise alignment with the chloroplast of *E. japonica* from China (MN159078), 424 SNPs (0.26%) and 809 INDELs (0.50%) were identified, displaying relatively large amount of intraspecific variations: larger than those of *Pyrus ussuriensis* (Cho et al. 2019), *Agrimonia pilosa* (Heo et al. 2020), *Camellia japonica* (Park et al. 2019b), and *Marchantia polymorpha* (Kwon et al. 2019). However, it is less than those of *Goodyera schlechtendaliana* (844 SNPs and 2045 INDELs; Oh et al. 2019b, 2019a) and *Gastrodia elata* (493 SNPs and 650 INDELs; Kang et al. 2020; Park et al. 2020) from Orchidaceae. Our chloroplast genome displays that direction of both LSC and SSC is reversely oriented compared with the chloroplast from China (MN159078), showing a structural variation within a species (Park et al. 2019a; Xi et al. 2019).

Seventy-two conserved genes from 11 chloroplast genomes including two *E. japonica* chloroplast genomes were included in phylogenetic analysis using the Bayesian inference using MrBayes v3.2.7a (Huelsenbeck and Ronquist 2001) and the maximum likelihood method using IQ-TREE v1.6.12 (Nguyen et al. 2015). The phylogenetic trees show that the Korean plant of *E. japonica* is sister to the Chinese accession of the species (Figure 1). Our phylogenetic analyses of chloroplast genomes confirm that *E*. *japonica* representing Crossosomatales is sister to the clade of Brassicales, Malvales, Huerteales, and Sapindales, consistent with previous studies (Soltis et al. 2011; Chase et al. 2016). Our chloroplast genome can be utilized to understand phylogeographic pattern of the warm temperate tree.

**Figure 1.**
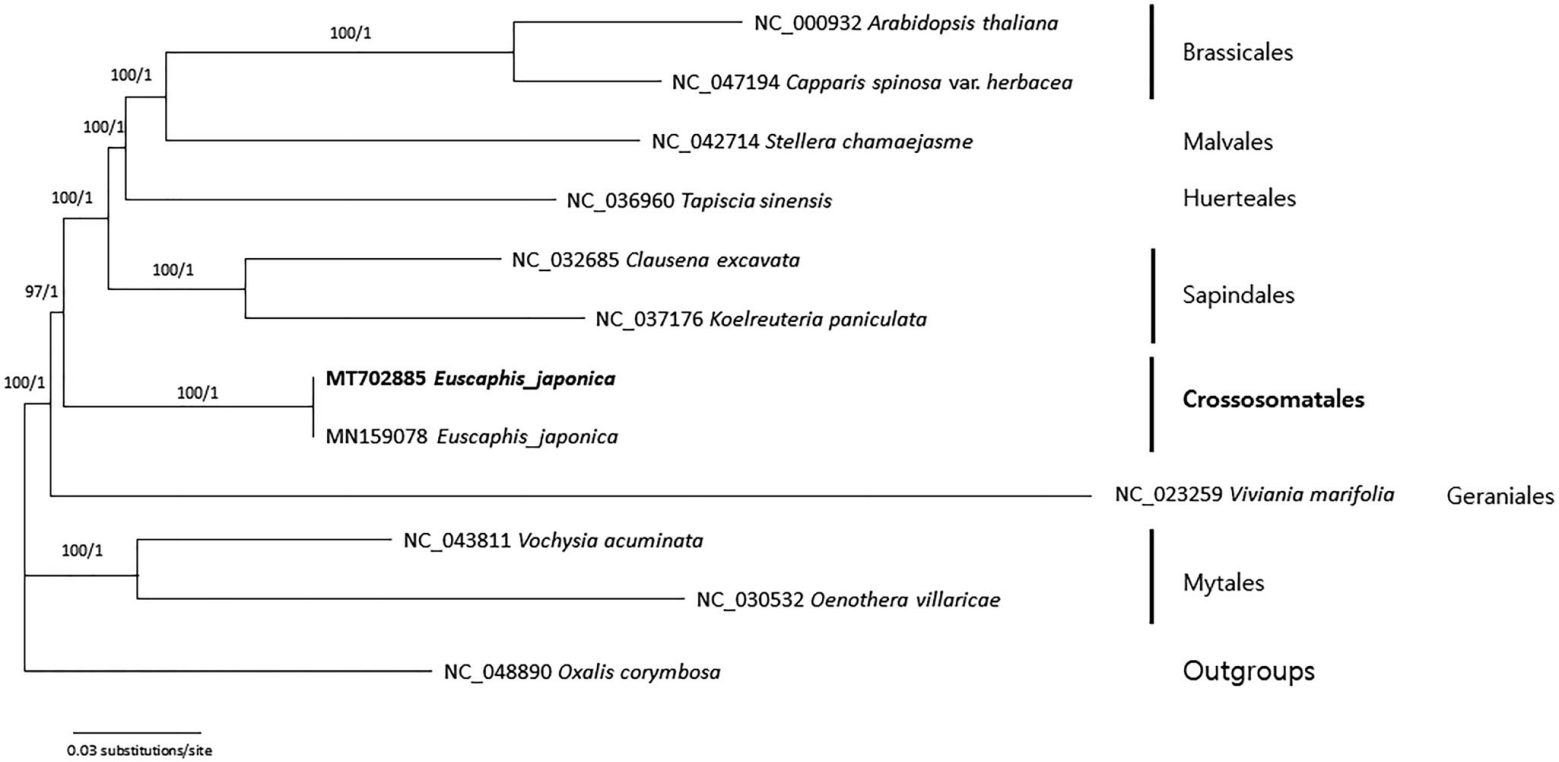
A maximum-likelihood tree from the analysis of 72 conserved genes, originated from two accessions of *Euscaphis japonica* (MT702885 in this study and MN159078), *Arabidopsis thaliana* (NC_000932), *Capparis spinosa* var. *herbacea* (NC_047194), *Clausena excavata* (NC_032685), *Koelreuteria paniculata* (NC_037176), *Oenothera villaricae* (NC_030532), *Stellera chamaejasme* (NC_042714), *Tapiscia sinensis* (NC_036960), *Vochysia acuminata* (NC_043811), and an outgroup species (*Oxalis corymbosa* (NC_048890)). The GTR model with gamma rates was used as a molecular model to construct the ML tree. A heuristic search was used with nearest-neighbor interchange (NNI) branch swapping, the Tamura-Nei model, and uniform rates among sites to construct maximum likelihood phylogenetic tree. All other options were set to their default values in IQ-TREE. Bootstrap analyses with 1000 pseudoreplicates were conducted with the same options. The same topology was produced from Bayesian inference. A Markov-chain Monte Carlo (MCMC) algorithm was employed for 1,000,000 generations, sampling trees every 200 generations, with four chains running simultaneously. Values above branches are bootstrap supports from the analysis of maximum likelihood and posterior probabilities. Names of orders followed APG IV (Chase et al. 2016).

## Data Availability

The chloroplast genome in this study is available in the NCBI GenBank (https://www.ncbi.nlm.nih.gov/genbank/) with an accession number, MT754180.
